# The Influence of Low Intensities of Light Pollution on Bat Communities in a Semi-Natural Context

**DOI:** 10.1371/journal.pone.0103042

**Published:** 2014-10-31

**Authors:** Aurelie Lacoeuilhe, Nathalie Machon, Jean-François Julien, Agathe Le Bocq, Christian Kerbiriou

**Affiliations:** 1 National Museum of Natural History, Ecology and Sciences Conservation Center, CESCO-UMR7204 MNHN-CNRS-UPMC, Paris, France; 2 Électricité de France S.A. (EDF), R & D, EPI Department, Moret sur Loing, France; University of Southern Denmark, Denmark

## Abstract

Anthropogenic light pollution is an increasingly significant issue worldwide. Over the past century, the use of artificial lighting has increased in association with human activity. Artificial lights are suspected to have substantial effects on the ecology of many species, e.g., by producing discontinuities in the territories of nocturnal animals. We analyzed the potential influence of the intensity and type of artificial light on bat activity in a semi-natural landscape in France. We used a species approach, followed by a trait-based approach, to light sensitivity. We also investigated whether the effect of light could be related to foraging traits. We performed acoustic surveys at sites located along a gradient of light intensities to assess the activity of 15 species of bats. We identified 2 functional response groups of species: one group that was light-tolerant and one group that was light-intolerant. Among the species in the latter group that appear to be disadvantaged by lighting conditions, many are rare and threatened in Europe, whereas the species from the former group are better able to thrive in disturbed habitats such as lighted areas and may actually benefit from artificial lighting. Finally, several methods of controlling light pollution are suggested for the conservation of bat communities. Recommendations for light management and the creation of dim-light corridors are proposed; these strategies may play an important role in protecting against the impact of light pollution on nocturnal animals.

## Introduction

Anthropogenic light pollution represents a growing global issue, currently affecting nearly 20% of the Earth's surface and increasing by approximately 6% per year [1]. It may have serious consequences for humans, animals and plants [2]. In the context of global changes, the energy currently allocated to artificial lighting could certainly be used differently to have a lower impact on biodiversity.

We assessed the impact of artificial light with the aim of contributing to better uses of light in view of the ecological needs of nocturnal species.

Light pollution commonly results from activities distributed over the entire geographical areas of developed countries. Indeed, artificially lit areas are not limited to cities but are generally associated with structures linked with urbanization, such as transportation networks, commercial and residential buildings and advertising spaces. Artificial lighting has a widespread influence on natural areas. At night, lights in cities, along roads or in industrial sites fragment the territories of nocturnal animals [2]. According to their ecological traits, bats are expected to be strongly influenced by artificial lighting. In Europe, all bats are nocturnal insectivores [3]. In addition, their prey -insects- is often concentrated near lights [4]. Thus, bats that feed on moths, and tolerate artificial light, such as *Pipistrellus pipistrellus,* may benefit from the amount of prey [5]. In contrast, species such as *Myotis spp.*, *Plecotus auritus* and *Rhinolophus hipposideros*, avoid lighted areas while commuting [6] and foraging [5]. Thus, artificial lights may intensify “interspecific competition for food” between rare species such as *R. hipposideros* and common species such as *P. pipistrellus* [7]. However, relatively few studies have focused on the impact of artificial lighting on bats at a community level [5], [8], [4], [9].

In addition, the attractiveness of lights to bats' prey is specific according to the type of light. For example, moths (an important prey category for certain bat species) are more attracted to mercury vapor lamps (white) than to low pressure sodium (orange) lamps [8].The activities of the bats at a given illuminated site depend on both the insect species (type of prey and abundance) attracted by the local type of light and the degree to which this light (intensity and type) repels various bat species. Consequently, the expected effects are not obvious and should be specific to each bat community.

Often perceived as an urban problem, light pollution is generally studied in urban areas, which are also associated with factors such as the noise characteristics of impervious substrates, noise pollution and air pollution. The effects of these urban factors could alter conclusions about the specific impact of light. We aimed to assess the influence of artificial light intensity and type on bat activity by performing an acoustic survey in a more natural context (a rural landscape) using both species and trait approaches. Indeed the potential impact of light on bats is largely unknown in a semi-natural context and may be species dependent [10], [9]. Given the declining status of bat populations throughout much of their European range [11], [12], a better understanding of the ecological needs of bat species is important for conservation purposes.

## Materials and Methods

### 1. Study area

The study was conducted in the Loire estuary in western France in a Natura 2000 site primarily composed of extensively managed land grazed by cattle (Corine Land Cover class: 231, “grassland”) and surrounded by hedgerows. Because our study consisted only of observations of bats without causing any disturbance to the animals, no permits were necessary. The 10, 000 ha study area consisted of isolated small unlighted rural residential areas and city centers that are generally lighted. An electrical power plant that was intensively lighted but covered less than 2% of the study area was also present in the area. Grasslands represent 96% and the Natura 2000 area 75% of the study area.

### 2. Sampling design and landscape analysis

To deal with the correlation between artificial surfaces and lighted areas, we employed a sampling design in which 119 stations with in a gradient of light intensities, were sampled, primarily at low light intensities (77.4% <5 lux, mean = 1.88±0.32 lux, minimum = 0 lux, maximum = 25 lux). Compared with standard levels of illuminance, such as 0.1 to 0.3 lux for a full moon under clear conditions and 15 lux for street lighting [13], our study investigated sites with medium intensity lighting. We recorded the lux level with a light meter (Chauvin Arnoux CA811) at the beginning of each sampling session.

The sampling stations were situated in a gradient of habitats (from natural grassland to discontinuous urban fabric) (spacing between the stations: mean = 3265.7 meters±239.06 (SE)) but were mostly located in semi-natural habitats that were subject to less intensive management. We assigned 200 meter circular buffers around each station and calculated the proportion of semi-natural habitat (grasslands, wetlands and woodlands) within the buffer using a regional land use database [14].

We sampled at different distances from hedgerows. We calculated the distance from each station to the nearest hedgerow (4 classes of distance: 0–24, 25–49, 50–99 and 100 or more meters from the hedgerow) using a regional hedgerow database [15].

### 3. Bat sampling

We sampled bats using standardized echolocation recordings at stations, a robust method for assessing the relationship between bat activities and the corresponding habitat [16]. We recorded echolocation calls using a Tranquility Transect Bat detector (Courtpan Design Ltd., Cheltenham, UK) with direct and continuous recording on a Zoom H2 digital recorder (Zoom Corporation, Tokyo, Japan) at a sampling rate of 96 ks/s in.wav format. We placed one detector at each station at 1.50 meters above the ground. Each station was sampled twice in 2011. The first session was conducted between June 15^th^ and July 31^st^. During this period, females give birth and suckle their offspring. A second session was performed between August 15^th^ and September 30^th^, when the young are flying and individuals are, most likely, less dependent on their natal roost. For each station and each session, we recorded one 30 minute sound sample. This sampling occurred randomly during the period of bat peak activity that begins 30 minutes after sunset and spans 4 hours [17]. It was only performed if weather conditions were favorable, i.e., no rain, wind speed lower than 10 km/h and temperature higher than 12°C. Nebulosity (i.e., cloudiness) (mean = 4.1±0.2 octas), temperature and wind speed data were retrieved from a local weather station [18]. To reduce the influence of weather conditions between sampled stations, we recorded 10 stations per night (5 stations simultaneously). Species calls were identified using Syrinx software version 2.6 [19] for spectrogram analyses and playback. Each contact was identified to the species level, except for *Plecotus austriacus* and *Plecotus auritus*, which were pooled in the *Plecotus spp.* group, and species from the *Myotis* genus due to their rarity and uncertainties in identification at the species level [20]. Note that from the perspective of foraging behavior, these species are primarily considered gleaners [3]. Because it was impossible to know the exact number of individuals foraging in the study areas, we instead used a bat activity measure, calculated as the number of calls per 30 minutes. Because this method does not allow individual monitoring, it was theoretically possible to detect the same individual at multiple sites. However, according to our sampling design, such events are rare and should not have biased our proxy measure of bat activity.

### 4. Statistical analysis

In a preliminary analysis, we attempted to distinguish the proportions of light generated by anthropogenic and natural sources. We determined the relationships between light intensity (response variable) and the following potential explanatory variables: (1) distance to the nearest town, (2) distance to the electric power plant, (3) nebulosity (0 to 8 octas), (4) time after sunset (in minutes) and (5) moon phase (0 to 58% visibility) for each sampling station using a generalized linear model (GLM with a quasi-Poisson error distribution). The results were evaluated using a type II ANOVA with an F-test (R package car [21]). Type II tests were calculated according to the principle of marginality, testing each term after all others, but ignoring the term's higher-order relatives. In this analysis and subsequent analyses, *P*-values were corrected for potential over-dispersion following Faraway [22].

The main analyses focused on the influence of light intensity and types (absence (no light mean = 0.2 lux±0.1); mercury vapor lamps (hereafter, white lamps) (white light intensity mean = 4.6 lux±1.2); and low-pressure sodium lamps (hereafter, orange lamps) (orange light intensity mean = 3.9 lux±1.2)) on each bat species' activity. The response variable was bat activity, and the explanatory variables were light characteristics. Among the explanatory variables we considered, the following co-variables well known to influence bat foraging activity [23] were incorporated: (1) date of sampling, (2) time after sunset, (3) temperature, (4) wind speed, (5) distance to the nearest hedgerow and (6) percentage of semi-natural habitat within the 200-meter buffer zone (light intensity and type effects were adjusted to these variables). To avoid multicollinearity, we systematically evaluated the correlations among continuous explanatory variables using Spearman's rho for quantitative variables [24]. We found no obvious correlations (Table A in File S1). To test the independence of light intensity (continuous variable) and light type (categorical variable), we used a Kruskal-Wallis test. The statistically significant test results (k = 60.12, P<0.001) indicated that light intensity and type could not be considered independent. Accordingly, we ran separate global models with either light intensity or light type and the 6 other explanatory variables. Moreover, we incorporated the spatial correlation structure into all our models using the expression x+y+x^2^+y^2^, where x and y are the geographic coordinates of the sampling stations (following the approach of Fortin and Dale [25], and Devictor et al., [26]). Based on the nature of the response variable (counts of bat calls), we expected a non-normal distribution. To identify the best model we built 4 GLMs for each tested species: one with a Poisson error distribution (GLMP), one with a negative binomial distribution (GLMNB) and 2 with a zero-inflated hypothesis (one with a Poisson error distribution (ZAP) and one with a negative binomial (ZANB) with the R package pscl [27]). The zero inflated models used were hurdle models (ZAP and ZANB) that consider presence and absence data (with a binomial function) and analyze the presence data in a second step with a count model (Poisson or a negative binomial) [28]. To identify the best error distribution, we used an AIC approach and examined the pattern of residuals, as proposed by Zuur et al. [28] (See in File S1 Tables B and C in File S1 for the AIC values and Tables D and E in File S1 for other variables). After the error distribution was identified (GLMP, GLMNB, ZAP, ZANB) for a species *i*, we ran separate global models (1) and (2),

1. [Bat activity]*_i_*∼light intensity+co-variables+spatial structure, [error distribution]*_i_*
2. [Bat activity]*_i_*∼light type+co-variables+spatial structure, [error distribution]*_i_*

where *i* denotes the bat species considered.

If the best model did not converge for both the intensity and type of light, we ran both models with a Poisson distribution.

**Figure 1:**
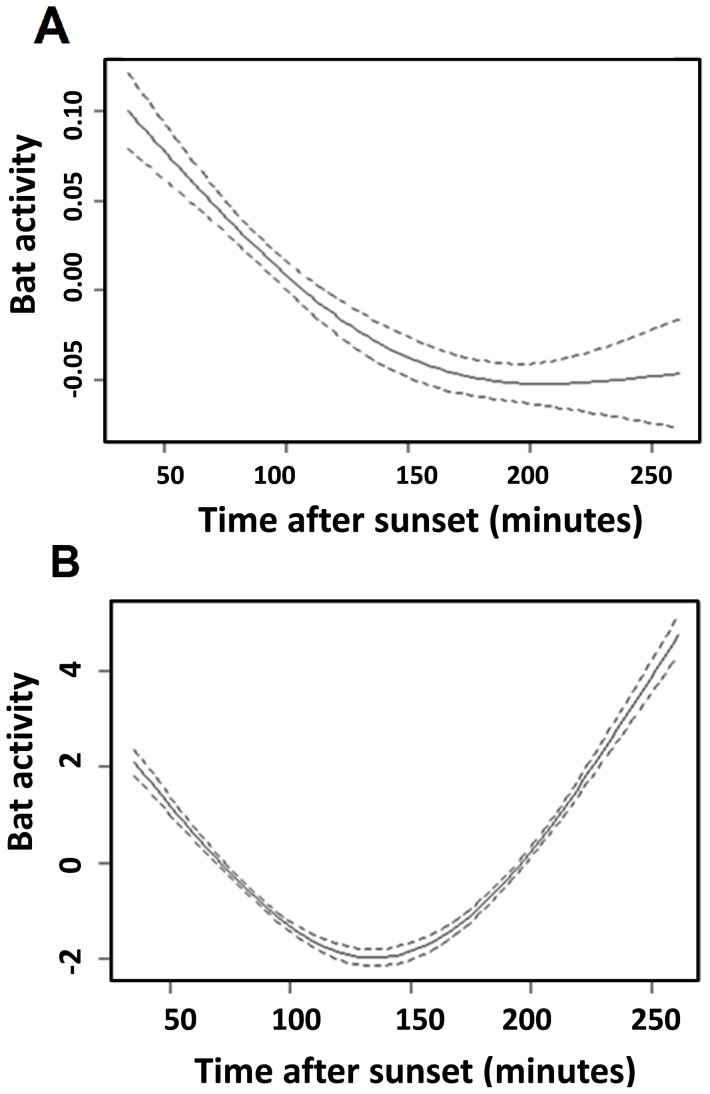
Effect of time after sunset on the activity of the tolerant group (A) and the intolerant group (B). Approximate significance of smoothing terms compared to linear effect: P<0.0001 for two groups; GAM (library mgcv). The y-axis is the value taken by the centered smooth. It is a relative measure of bat activity (relative numbers of calls). It is the contribution (at a value of the covariate) made to the fitted value for that smooth function.

In a final analysis, we studied the relationship between the trait “artificial light sensitivity” and the pattern of change in bat activities during the night. Bat species were classified according to their sensitivity to light intensity based upon our study, i.e., tolerant bats, for which activity was most likely positively influenced by light intensity *vs.* intolerant bats, for which activity appeared to be negatively influenced by light intensity. Species that showed no significant trend were excluded from this analysis. Because we expected a nonlinear effect of time after sunset, we used a general additive model (GAM) with time after sunset as the smoothed term [29], [28]. The fixed effects were the same as those found in the previous analysis with GLM (Table F in File S1). We reported the numerical results of the GLM and plotted the GAM (Figure 1) (R package mgcv) [30]. All analyses were performed with R version 2.13.0 [31].

## Results

### 1. Assessment of bat activity

We recorded 205,036 calls belonging to 15 species. In particular, we obtained a large amount of data for 7 species (from at least 20 stations) (Table 1). All of the species recorded during the reproductive period (the first survey period) were also found during the second period. However, certain species, e.g., *Barbastella barbastellus*, were only detected during the second period (August–September). The most abundant species in both periods was *Pipistrellus pipistrellus* (Table 1).

**Table 1:** Observed species and their abundances.

*Species*	*Percentage of stations at which bats were detected*		*Mean number of calls per minute in occupied stations*	
	*1st period*	*2nd period*	*1st period*	*2nd period*
*Barbastella barbastellus*	0	7.4	0	1.7
*Eptesicus serotinus*	8.4	17.0	3.9	2.8
*Pipistrellus kuhlii*	38.9	59.6	5.9	12.0
*Pipistrellus nathusii*	49.5	62.8	4.5	6.5
*Pipistrellus pipistrellus*	80.0	86.2	13.3	8.7
*Pipistrellus pygmaeus*	2.1	3.2	0.2	1.2
*Nyctalus leisleri*	7.4	12.8	2.3	0.9
*Nyctalus noctula*	10.5	9.6	1.2	0.8
*Myotis spp.* *	3.1	12.6	1.6	4.8
*Plecotus spp.* **	7.4	3.2	1	1.2

The percentages are calculated based on the number of stations at which at least one bat species call was recorded relative to the total number of sampling stations. The mean number of calls per minute was calculated only for the sampling stations at which at least one call was recorded. For *Myotis* and *Plecotus* spp., the counts are given at the genus level.

* *Myotis bechsteinii*, *Myotis daubentonii*, *Myotis myotis*, *Myotis mystacinus*, *Myotis nattereri*.

**Plecotus austriacus and *Plecotus auritus*.

### 2. Proportions of light explained by anthropogenic and natural sources

The distance to the industrial site, distance to the nearest town, time after sunset and nebulosity had a significant influence on light intensity. In our sampling, artificial sources of light were responsible for the majority of the explained variance: 45% was explained by the distance to the nearest town, 22% by the distance to the industrial site, 18% by the nebulosity, 14% by the time after sunset and 1.5% by the moon phase (not a significant effect) (Table 2).

**Table 2:** Effects of different factors on the light intensity at the sampling stations.

Variables	SS	F	Pr (>F)
Distance to the industrial area	44.99	5.423	0.020
Distance to the nearest town	93.26	11.241	0.001
Nebulosity	36.39	4.386	0.038
Time after sunset	28.08	3.379	0.068
Moon phase	3.15	0.379	0.539

Results of the GLM. SS is the sum of squares, F is the mean of squares for the factor/mean of squares for the error, and Pr is the probability value associated with the test (p-value of Anova).

### 3. The effect of light intensity on bat species activity

We observed a significant positive effect of light intensity on the activity of *Pipistrellus pipistrellus*, *Pipistrellus pygmaeus*, *Pipistrellus kuhlii*, *Eptesicus serotinus* and *Nyctalus noctula*, a significant negative effect for *Nyctalus leisleri*, *Myotis spp.* and *Plecotus spp.* and no detectable effect on *B. barbastellus* or *Pipistrellus nathusii* (Table 3). The effects of the other variables are reported in Table D in File S1.

**Table 3:** Influence of light intensity on the activity of each bat species.

Species	Selected model	Estimated effect of light intensity	P-value	Foraging strategy [3]
*Barbastella barbastellus*	Zero inflated model with negative binomial-count model	*β* = −7.21±81.06 SE	*P = 0.93*	*Aerial hawking*
*Eptesicus serotinus*	Zero inflated model with negative binomial-count model	*β* = 6.67±1.17 SE	*P<0.001*	*Aerial hawking*
*Pipistrellus kuhlii*	Zero inflated model with negative binomial-count model	*β* = 0.15±0.087 SE	*P = 0.076*	*Aerial hawking*
*Pipistrellus nathusii*	Zero inflated model with negative binomial-count model	*β* = 0.092±0.066 SE	*P = 0.16*	*Aerial hawking*
*Pipistrellus pipistrellus*	Negative binomial distribution	*β* = 0.074±0.037 SE	*P = 0.046*	*Aerial hawking*
*Pipistrellus pygmaeus*	Zero inflated model with Poisson distribution-count model	*β* = 1.95±0.21 SE	*P<0.001*	*Aerial hawking*
*Nyctalus leisleri*	Zero inflated model with negative binomial-count model	*β* = −6.77±2.61 SE	*P = 0.0097*	*Aerial hawking*
*Nyctalus noctula*	Zero inflated model with Poisson distribution-count model	*β* = 0.50±0.07 SE	*P<0.001*	*Aerial hawking*
*Myotis spp.*	Negative binomial distribution	*β* = −5.98±2.35 SE	*P = 0.011*	*Gleaner*
*Plecotus spp.*	Zero inflated model with Poisson distribution-count model	*β* = −12.62±2.04 SE	*P<0.001*	*Gleaner*

[3] Dietz C., Helversen O. von, Nill D.(2009) L'encyclopédie des chauves-souris d'Europe et d'Afrique du Nord: Biologie, caractéristiques, protection. Delachaux et Niestlé, Paris. 400 p.

### 4. The effect of light type on bat species activity

We observed that white light had a significant positive effect on *P. pipistrellus* and *P. kuhlii* and a significant negative effect on *N. noctula* (Table 4).

**Table 4:** Influence of light type on the activity of each bat species.

Species	Selected model	Estimated effect of type of light	Back-transformed estimate effects		P-value
*Barbastella barbastellus*	Poisson distribution		Absence	1.22	
		White *β* = −17.52±3580.43	White	−16.3	*P = 1.00*
		Orange *β* = −0.48±1.58	Orange	0.74	*P = 0.76*
*Eptesicus serotinus*	Zero inflated model with negative binomial-count model		Absence	24.68	
		White *β* = −0.71±1.10	White	23.97	*P = 0.52*
		Orange *β* = −7.29±1.28	Orange	17.39	*P<0.001*
*Pipistrellus pipistrellus*	Negative binomial distribution		Absence	3.82	
		White *β* = 2.45±0.74	White	6.27	*P<0.001*
		Orange *β* = 1.72±0.67	Orange	5.55	*P = 0.01*
*Pipistrellus pygmaeus*	Poisson distribution		Absence	−3.11	
		White *β* = −14.19±22.28.10^2^	White	−17.3	*P = 0.99*
		Orange *β* = 4.78±1.88	Orange	1.67	*P = 0.01*
*Pipistrellus kuhlii*	Zero inflated model with negative binomial-count model		Absence	−1.31	
		White *β* = 5.33±1.33	White	4.02	*P<0.001*
		Orange *β* = 2.87±0.98	Orange	1.56	*P = 0.003*
*Pipistrellus nathusii*	Zero inflated model with negative binomial-count model		Absence	3.36	
		White *β* = 0.99±0.73	White	4.36	*P = 0.17*
		Orange *β* = 0.42±0.56	Orange	3.78	*P = 0.46*
*Nyctalus leisleri*	Zero inflated model with negative binomial-count model		Absence	−2.52	
		White *β* = 1.22±2.07	White	−1.30	*P = 0.56*
		Orange *β* = −0.97±1.47	Orange	−3.49	*P = 0.51*
*Nyctalus noctula*	Zero inflated model with Poisson distribution-count model		Absence	25.29	
		White *β* = −3.58±1.81	White	21.70	*P = 0.05*
		Orange *β* = 2.23±1.95	Orange	27.52	*P = 0.25*
*Myotis spp.*	Poisson distribution		Absence	0.19	
		White *β* = 3.69±1.80	White	3.88	*P = 0.05*
		Orange *β* = 0.23±3.66	Orange	0.42	*P = 0.95*
*Plecotus spp.*	Poisson distribution		Absence	−3.15	
		White *β* = −17.15±26.79.10^+2^	White	−20.3	*P = 0.99*
		Orange *β* = −1.18±1.95	Orange	−4.33	*P = 0.54*

Because the type of light is a categorical variable, the given estimate is the average estimate of bat activity for each type (white or orange) compared with the absence of light. Thus, the p-value provides information about the significance of the difference between an absence of light *vs.* the artificial light type (white or orange). Back-transformed estimate effects represent the average estimate of bat activity for each color type on the original scale.

Orange light had a significant positive effect on *P. pipistrellus* and *P. kuhlii* and a significant negative effect on *E. serotinus* (Table 4). We are cautious about the conclusions for the other species, because the effect could not be assessed using the best model. For the effects of the other variables, e.g., weather conditions, see Table E in File S1.

### 5. Comparison between light intensity and type effects

Intensity and light types were correlated. Accordingly, they were tested using two separate models, but the models were identical in the other variables included and the error distribution. We used the AIC to identify the best predictor (light intensity *vs.* type). We concluded that type of light better explained our data than light intensity for *P. pipistrellus*, *P. pygmaeus*, *P. kuhlii*, and *Myotis spp.*, whereas for *E. serotinus*, *N. noctula*, *N. leisleri*, and *Plecotus spp.* light intensity appeared to be the best predictor (Table 5 and see Table C in File S1). Note that for *P. pygmaeus*, *Myotis spp.* and *Plecotus spp.*, the results must be interpreted cautiously because the comparison between light intensity and type of light could not be performed using the best model, but was based on a model with a Poisson distribution. Moreover, we reached no conclusions for *P. nathusii and B. barbastellus* because light intensity and light type were not identified as significant explanatory variables.

**Table 5:** Comparison between models using light intensity or light type as explanatory variables.

Species	Model	AIC of model testing influence of light intensity on bat activity	AIC of model testing influence of light type on bat activity
Barbastella barbastellus*	GLMP	1380	**1243**
*Eptesicus serotinus*	ZANB	**244**	259
*Pipistrellus pipistrellus*	GLMNB	1794	**1541**
Pipistrellus pygmaeus*	GLMP	1119	**1004**
*Pipistrellus kuhlii*	ZANB	1103	**1033**
*Pipistrellus nathusii*	ZANB	1385	**1258**
*Nyctalus leisleri*	ZANB	**191**	270
*Nyctalus noctula*	ZAP	**193**	201
Myotis ssp.*	GLMP	2019	**1800**
Plecotus ssp.*	GLMP	**525**	1021

. AIC values are given for each model. (GLMP) indicates a Poisson distribution, (GLMNB) a negative binomial distribution, (ZAP) a zero inflated model with a Poisson distribution and (ZANB) Zero inflated model with negative binomial distribution. (*) indicates that as the best model did not converge for both effect (light intensity and light type), we compared the two models using a Poisson error distribution. The models retained based on the smallest AIC value are shown in bold [45].

### 6. The effect of time of night on the activity of bat species

Based on the observed effects of light intensity on the activity of bat species, we sorted species into two groups: light-tolerant *vs.* light-intolerant species (Table 3). Two major and non exclusive foraging strategies were used by bats in our study. Certain species, hereafter designated aerial hawkers, are primarily open space foragers; they capture flying prey. Others, hereafter designated gleaners, usually capture their prey from substrates in cluttered environments, although they may also capture flying prey [3], [32], [33]. These foraging groups are not absolute categories. The light-intolerant group included bat species with different foraging strategies [3]: aerial hawking bats (*N. leisleri*) and primarily gleaning bats (*Myotis spp.* and *Plecotus spp.*), whereas the tolerant group did not include any gleaning bats. For each group, we tested the influence of sampling time (number of minutes after sunset) on bat activity. We found a significant negative effect of time of night on the activity of the light-tolerant group: β = −5.14.10^−4^±8.01.10^−5^ SE, z-value = −6.43, P-value <0.001. We found a positive effect on the light-intolerant group: β = 8.59.10^−3^±9.26.10^−4^ SE, z-value = 9.28, P-value <0.001. The light-tolerant bats were more active during the early night than the light-intolerant bats, which were more active later in the night. Note, however, that non-linear effects were detected for the light-intolerant bats, whose activity shows a peak at the beginning of the night. This peak may reflect a transit activity from the roost to foraging areas (Figure 1).

## Discussion

Our purpose was to understand the influence of artificial light on bats in a semi-natural context with two levels of analysis. Using a species approach, we showed that light intensity and type had different effects on bat species. Then, using a trait-based approach, we showed that the effects appeared to be differentiated based on bat foraging type.

### 1. Different effects of light intensity on different bat species

The most plausible hypothesis to explain the attraction of certain bats to light is that the halo of artificial light offers greater [8] and more predictable prey availability than dark sites [10]. The increased density of moths around lights allows bats to feed more efficiently and to reduce their hunting time [34]. However, the most plausible hypotheses to explain the avoidance of lighted areas by certain bat species are that foraging activity in areas with artificial light could increase predation risk [35] or that the orientation abilities of the animals may be negatively affected by artificial lighting [36]. Our results (Table 3) identified two groups of species that were differentially influenced by light intensity. Five species appeared to be attracted by light (*P. pipistrellus*, *P. pygmaeus*, *P. kuhlii*, *E. serotinus* and *N. noctula*), whereas other taxa seemed to be negatively affected by light (*N. leisleri*, *Myotis spp.* and *Plecotus spp.*) (Table 1). Interestingly, among the species we studied, the response to light appears to be associated with a specific foraging strategy; the light-tolerant species are all aerial hawkers. Our results are consistent with the few studies published on this topic [5], [9]. For two species (*B. barbastellus* and *P. nathusii*), we were unable to detect any significant effect of light intensity. In the case of *B. barbastellus*, it is probable that this result was due to the small sample size. However, in the case of *P. nathusii*, one of the most abundant species, we did not detect any obvious attraction to or avoidance of light when our study included the main foraging habitats of this species (wetlands, woodlands, and in late summer during migration, urban areas [3]).

### 2. The effects of light on tolerant species

According to the non-independence of light intensity and light type (the stations with white light exhibited a higher light intensity, on average, than the stations with orange light), we could have expected that 1) the intolerant species avoided white light, whereas 2) the tolerant species were attracted by white light. However, this was not always the case. The second hypothesis was verified for *P. pipistrellus* and *P. kuhlii* but not for *E. serotinus* and *N. noctula*. We argue that to explain these results, we must consider how bats and their prey perceive the spectra.

Four tolerant species are widely distributed and considered common in France [37] and Europe [38]. According to the literature, these species forage in a variety of habitats, especially urbanized areas (*E. serotinus* [39]; *P. pygmaeus* [40]; *P. pipistrellus* and *P. kuhlii* [37]). At present, the majority of bats in urban areas of Western Europe are thought to be *P. pipistrellus* [8]. However, the fifth tolerant species, *N. noctula*, is classified as “Near Threatened” [12]. This species usually flies above streetlights but sometimes flies in light beams [10]. Our results showed that the activity of *N. noctula* was negatively influenced by vapor lamps (i.e., white light) but yielded a non-significant result for low-pressure sodium lamps (i.e., orange light), in contrast with *P. pipistrellus* and *P. kuhlii*, which were positively influenced by both types of lights. Moreover, the activity of these two species appeared to be better explained by light type than by light intensity. The most common foragers within cities are bats that are known to benefit from white streetlamps [41]. Nevertheless, the effect of light type on bats is not obvious. The activity of *E. serotinus* was only negatively influenced by low pressure sodium lamps; a non-significant result was found for vapor lamps. There was no effect of light type for all other species (Table 4). Actually, roads with vapor lamps attract more foraging bats than roads lighted by low-pressure sodium lamps or unlighted roads [8], and vapor lamps attract more moths than low-pressure sodium lamps [4]. Moreover, lights have a negative effect on the defensive escape behavior response of moths to bat echolocation calls [34]. Therefore, the presence of insects around artificial lights may have a greater effect on bat activities than does light type; the spectrum of the lamps may be producing an indirect effect on the bats.

### 3. The effects of light on intolerant species

Most of the species we detected were potentially disadvantaged by light are of conservation concern. For example, two of *Myotis* species whose calls we identified were species (*M. bechsteinii* and *M. myotis*) classified in Annex II of the Habitat Directive of the European Union (92/43/EEC), and, according to the IUCN Red List, two species are classified as “Near Threatened” in France (*M. bechsteinii* and *N. leisleri*). Furthermore, in Europe, light-intolerant species are primarily found foraging in semi-natural habitats such as woodlands, pastures or wetlands. However, although our results are congruent with the information about these species in the literature, we collected few data for these rare species.

### 4. Different foraging strategies for light-intolerant and light-tolerant bat species at night

Flying insect biomass peaks during the crepuscular period [3]. Our results (Figure 1) are consistent with the findings of Gaisler et al. [41] and Rydell et al. [42] that light-tolerant bats were the most active during the first minutes after sunset and that their activity declined thereafter, when the availability of most insects also declined [42].The activity of light-intolerant species was low in the first minutes after sunset and increased thereafter. The tolerant group is adapted to hunting during twilight and they are more rapid fliers than other bats. In contrast, the activity of the light-intolerant group decreased in the first minutes after sunset and increased later during the night (Figure 1). Because the bats belonging to this group are disturbed by light, they could be at a disadvantage. The intolerant group forages in darkness, where prey availability could be reduced by artificial lights that attract insects. Thus, artificial lighting could have a negative impact on certain species.

We found that groups are specialized for different prey. The tolerant group primarily hunts flying insects near light (e.g., moths) [34], whereas the gleaners of the intolerant group primarily hunt non-flying insects [3]. Street lighting can alter the composition of ecosystems by attracting specific invertebrate communities [43]. Additionally, bat foraging activity follows the nocturnal phenology of insects [42]. For many gleaners, such as many *Myotis* species, which are slower fliers, “early emergence would probably not result in much extra benefit but only in added cost” and “would result in higher predation risk at the higher light level” [42]. Indeed, Jones and Rydell [44] hypothesize a trade-off between reduced predation risk and increased foraging efficiency. These preliminary findings could be extended by surveys throughout the entire night to study foraging phenology near colonies.

### 5. Interspecific competition

The particular phenology of the tolerant bats and their opportunism could explain their ability to adapt to and take advantage of artificial lighting. Due to this trait, artificial lighting could generate interspecific competition by making certain prey of the light-intolerant bats available to tolerant bats such as *Pipistrellus spp.* as suggested by Arlettaz et al. [7].

### 6. Implications for light management and bat conservation planning

We conclude that the majority of the bat species that we examined in this study appear to be sensitive to artificial light and that the activity of bats is less in the lighted areas, even in semi-natural habitats. Artificial light, even low-intensity light, appeared to be sufficiently strong to disturb certain bat species. The tolerant bats may be better able to thrive in disturbed habitats, such as lighted areas, and these species actually appeared to be more abundant at the study site (Table 1).

To better preserve bat communities, we propose several complementary measures: light intensity could be reduced in the early night, and the timing of lighting could be restricted subject to security concerns. Moreover, to reduce the “trespass” of lighting [13], lights should be designed to illuminate only their target areas installed at lower heights and with a controlled orientation. Reflective surfaces could be replaced by light-absorbent ones.

We did not detect any significant effect of light type on intolerant bat activity whereas several studies have found that species from the intolerant group appeared to be affected by several types of light including mercury vapor [5], sodium [6] and white Monaro LED lights [9]. We also showed that two tolerant species are disturbed by orange (*E. serotinus*) and white lights (*N. noctula*). Thus, the type and intensity of the emitted light cannot be overlooked. Low-pressure sodium lights or filters for mercury lights could be used.

Meadows, wetlands, riparian habitats and woodlands are the habitats that are the most frequented by bats [3]. Thus, it is particularly important to decrease light pollution in these habitats, where artificial light can be particularly harmful to light-intolerant bats. Light pollution may play a role in the fragmentation of bat foraging territories by interrupting commuting routes [6] and limiting foraging habitats for certain intolerant species. Overlaying the “nocturnal network” (an area without artificial lights) with a classical network based on natural patches and their associated corridors could be both interesting and necessary for the protection of nocturnal animals, including bats against the impacts of light pollution and for contributing to environmental awareness.

## Supporting Information

File S1 **File contains six supporting tables.** **Table A:** Tests of independence between variables. **Table B:** Influence of light intensity on bat activity: AIC of each model type for each detected species. *Selection between 4 models: GLM with Poisson distribution (Poisson), GLM with negative binomial distribution (Negative binomial), Zero inflated model with Poisson distribution (ZAP) and Zero inflated model with negative binomial distribution (ZANB).Given in bold letters are the retained models according to the smallest AIC value* [*45*] *(/the model did not converge).* **Table C:** Influence of light type on bat activity: AIC of each model type for each detected species. *Selection between 4 models: GLM with Poisson distribution (Poisson), GLM with negative binomial distribution (Negative binomial), Zero inflated model with Poisson distribution (ZAP) and Zero inflated model with negative binomial distribution (ZANB). Given in bold letters are the retained models according to the smallest AIC value* [*45*] *(/the model did not converge. in bold and underlined when the AIC value is smaller than with light intensity).* **Table D:** Effects of light intensity, weather, spatial and landscape conditions and date on the activity of each bat species. **Table E:** Effects of light type, weather, spatial and landscape conditions and date on the activity of each bat species. **Table F:** Effects of light intensity, weather, spatial and landscape conditions and date on the activity of each group (GLM results).
(DOC)

## References

[1] Hölker F, Wolter C, Perkin EK, Tockner K (2010) Light pollution as a biodiversity threat. Trends in Ecology & Evolution 25: 681–682. 21035893 10.1016/j.tree.2010.09.007

[2] Rich C, Longcore T (Eds.) (2006) Ecological Consequences of Artificial Night lighting. Island Press, Washington, D.C. 458 p.

[3] Dietz C, von Helversen O, Nill D (2009) L'encyclopédie des chauves-souris d'Europe et d'Afrique du Nord: Biologie, caractéristiques, protection. Delachaux et Niestlé, Paris. 400p.

[4] Rydell J, Baagoe HJ (1996) Street lamps increase bat predation on moths. Entomologisk Tidskrift 117: 129–135.

[5] Rydell J (1992) Exploitation of insects around streetlamps by bats in Sweden. Functional Ecology 6: 744–750.

[6] Stone EL, Jones G, Harris S (2009) Street lighting disturbs commuting bats. Current Biology 19: 1123–1127. 19540116 10.1016/j.cub.2009.05.058

[7] Arlettaz R, Godat S, Meyer H (2000) Competition for food by expanding pipistrelle bat populations (*Pipistrellus pipistrellus*) might contribute to the decline of lesser horseshoe bats (*Rhinolophus hipposideros*). Biological Conservation 93: 55–60.

[8] Blake D, Hutson AM, Racey PA, Rydell J, Speakman JR (1994) Use of lamplit roads by foraging bats in southern England. Journal of Zoology 234: 453–462.

[9] Stone EL, Jones G, Harris S (2012) Conserving energy at a cost to biodiversity? Impacts of LED lighting on bats. Global Change Biology 18: 2458–2465.

[10] Rydell J (2006) Bats and Their Insect Prey at Streetlights. In: Rich C. and Longcore T. editors. Ecological Consequences of Artificial Night Lighting. Island Press, Washington, D.C. pp. 43–60.

[11] Stebbings R (1988) The Conservation of European Bats. Christopher Helm, London, UK. 256 p.

[12] IUCN (2012) The IUCN Red List of Threatened Species. Version 2012.2. Available: http://www.iucnredlist.org.

[13] Gaston KJ, Bennie J, Davies TW, Hopkins J (2013) The ecological impacts of nighttime light pollution: a mechanistic appraisal. Biological reviews 88: 912–927. 23565807 10.1111/brv.12036

[14] CORELA (2007) Land use database. Available: http://www.corela.org/fondsdocumetaires/cartesoccupationdusol.html. Accessed 2012 Jun.

[15] CORELA (2007) Hedgerows database. Available: http://www.corela.org/fondsdocumetaires/cartesbocages.html. Accessed 2012 Jun.

[16] Stahlschmidt P, Brühl CA (2012) Bats as bioindicators - the need of a standardized method for acoustic bat activity surveys. Methods in Ecology and Evolution 3: 503–508.

[17] Roche N, Catto C, Langton S, Aughney T, Russ J (2005) Development of a Car-Based Bat Monitoring Protocol for the Republic of Ireland. Irish Wildlife Manuals, No. 19. National Parks and Wildlife Service, Department of Environment, Heritage and Local Government, Dublin, Ireland.

[18] Météo France (2012) French national meteorological service. Database available: www.meteofrance.com. Accessed 2012 Aug.

[19] Burt J (2006) Syrinx a software for real time spectrographic recording, analysis and playback of sound. [http:/www.syrinxpc.com].

[20] Russ J (1999) The Bats of Britain and Ireland: Echolocation Calls, Sound Analysis and Species Identification. Alana Books, Bishop's Castle. 103 p.

[21] Fox J, Weisberg S (2011) An {R} Companion to Applied Regression, Second Edition. Thousand Oaks CA: Sage. Available: http://socserv.socsci.mcmaster.ca/jfox/Books/Companion

[22] Faraway JJ (2006) Extending the linear model with R, Generalized linear, mixed effects and nonparametric regression models. Chapman & Hall/CRC, USA. 312p.

[23] Scanlon AT, Petit S (2008) Effects of site, time, weather, and light on urban bat activity and richness: considerations for survey effort. Wildlife Research 35: 821–834.

[24] Crawley MJ (2009) The R book. John Wiley & Sonc, Chicago, USA.

[25] Fortin M, Dale M (2005) Spatial analysis. A guide for ecologists. Cambridge University Press, Cambridge, England. 365 p.

[26] Devictor V, Julliard R, Jiguet F (2008) Distribution of specialist and generalist species along spatial gradients of habitat disturbance and fragmentation. Oikos 117: 507–514.

[27] Jackman S (2012) pscl: Classes and Methods for R Developed in the Political Science Computational Laboratory, Stanford University. Department of Political Science, Stanford University. Stanford, California. R package version 1.04.4. Available: http://pscl.stanford.edu/.

[28] Zuur AF, Ieno EN, Walker NJ, Saveliev AA, Smith GM (2009) Mixed effects models and extensions in ecology with R. Springer, New York, 574 p.

[29] Guisan A, Edwards TC, Hastie JT (2002) Generalized linear and generalized additive models in studies of species distribution: setting the scene. Ecol Modell 157: 89–100.

[30] Wood SN (2006) Generalized additive models: an introduction with R. Chapman and Hall/CRC. 410 p.

[31] R Development Core Team (2011) R: A language and environment for statistical computing. R Foundation for Statistical Computing, Vienna, Austria. [http://www.R-project.org/]

[32] Fenton MB, Bogdanowicz W (2002) Relationships between external morphology and foraging behaviour: bats in the genus Myotis. Canadian Journal of Zoology 80: 1004–1013.

[33] Schnitzler HU, Kalko EK (2001) Echolocation by Insect-Eating Bats We define four distinct functional groups of bats and find differences in signal structure that correlate with the typical echolocation tasks faced by each group. Bioscience 51: 557–569.

[34] Acharya L, Fenton MB (1999) Bat attacks and moth defensive behavior around street lights. Can J Zool 77: 27–33.

[35] Rydell J, Speakman JR (1995) Evolution of nocturnality in bats. Potential competitors and predators during their early history. Biol J Linn Soc 54: 183–191.

[36] McGuire LP, Fenton MB (2010) Hitting the wall: light affects the obstacle avoidance ability of free-flying little brown bats (*Myotis lucifugus*). Acta Chiropterologica 12: 247–250.

[37] Arthur L, Lemaire M (2009) Les chauves-souris de France, Belgique, Luxembourg et Suisse. Biotope, Mèze, Muséum National d'Histoire Naturelle. Paris. 544p.

[38] Mitchell-Jones AJ, Amon G, Bogdanowicz W, Krystufek B, Reijnders PJH, et al. (1999) The atlas of European mammals. Academic Press, London. 496 p.

[39] Catto CMC, Hutson AM, Racey PA, Stephenson PJ (1996) Foraging behavior and habitat use of serotine bat (*Eptesicus serotinus*) in southern England. J Zool Lond 238: 623–633.

[40] Rainho A (2007) Summer foraging habitats of bats in a Mediterranean region of the Iberian Peninsula. Acta Chiropterologica 9: 171–181.

[41] Gaisler J, Zukal J, Rehak Z, Homolka M (1998) Habitat preference and flight activity of bats in a city. Journal of Zoology 244: 439–445.

[42] Rydell J, Entwistle A, Racey PA (1996) Timing of foraging flights of three species of bats in relation to insect activity and predation risk. Oikos 76: 243–252.

[43] Davies TW, Bennie J, Gaston KJ (2012) Street lighting changes the composition of invertebrate communities. Biology Letter 8: 764–767. 10.1098/rsbl.2012.0216 PMC3440964 22628095

[44] Jones G, Rydell J (1994) Foraging Strategy and Predation Risk as Factors Influencing Emergence Time in Echolocation Bats. Philosophical Transactions: Biological Sciences 346: 445–455.

[45] Burnham KP, Anderson DR (2002) Model selection and multimodel inference: a practical information - theoretic approach. Second edition ed. Springer-Verlag, New York. 488p.

