# Supplementary material for: The Influence of Low Intensities of Light Pollution on Bat Communities in a Semi-Natural Context
Source: PLoS One. 2014 Oct 31;9(10):e103042. doi: 10.1371/journal.pone.0103042 (PMC4215844; doi:10.1371/journal.pone.0103042)
Supplement: File S1 — File contains six supporting tables. Table A: Tests of independence between variables. Table B: Influence of light intensity on bat activity: AIC of each model type for each detected species. Selection between 4 models: GLM with Poisson distribution (Poisson), GLM with negative binomial distribution (Negative binomial), Zero inflated model with Poisson distribution (ZAP) and Zero inflated model with negative binomial distribution (ZANB).Given in bold letters are the retained models according to the smallest AIC value [45] (/the model did not converge). Table C: Influence of light type on bat activity: AIC of each model type for each detected species. Selection between 4 models: GLM with Poisson distribution (Poisson), GLM with negative binomial distribution (Negative binomial), Zero inflated model with Poisson distribution (ZAP) and Zero inflated model with negative binomial distribution (ZANB). Given in bold letters are the retained models according to the smallest AIC value [45] (/the model did not converge. in bold and underlined when the AIC value is smaller than with light intensity). Table D: Effects of light intensity, weather, spatial and landscape conditions and date on the activity of each bat species. Table E: Effects of light type, weather, spatial and landscape conditions and date on the activity of each bat species. Table F: Effects of light intensity, weather, spatial and landscape conditions and date on the activity of each group (GLM results). (DOC) [file pone.0103042.s001.doc]

**Supporting Information**

Table A: Tests of independence between variables.

|  | *Date* | *Temperature* | *Wind* | *Nebulosity* | *Time after sunset* | *Semi-natural area* | *Distance to hedgerow* | *Distance to industrial area* | *Distance to the nearest town* | *Light intensity* | *Moon appearance* |
| --- | --- | --- | --- | --- | --- | --- | --- | --- | --- | --- | --- |
| Date | 1.000 | 0.079 | 0.246 | -0.085 | -0.095 | -0.026 | -0.043 | 0.173 | 0.040 | 0.041 | -0.117 |
| Temperature | 0.079 | 1.000 | -0.164 | 0.037 | 0.180 | -0.316 | -0.013 | 0.019 | -0.072 | 0.018 | 0.142 |
| Wind | 0.246 | -0.164 | 1.000 | -0.183 | 0.518 | -0.028 | 0.106 | -0.147 | 0.186 | -0.003 | -0.182 |
| Nebulosity | -0.085 | 0.037 | -0.183 | 1.000 | 0.088 | 0.010 | 0.017 | 0.115 | 0.068 | -0.096 | 0.101 |
| Time after sunset | -0.095 | 0.180 | 0.518 | 0.088 | 1.000 | -0.084 | 0.032 | -0.143 | 0.049 | 0.036 | -0.153 |
| Semi-natural area | -0.026 | -0.316 | -0.028 | 0.010 | -0.084 | 1.000 | -0.212 | -0.030 | -0.007 | 0.028 | -0.175 |
| Distance to hedgerow | -0.043 | -0.013 | 0.106 | 0.017 | 0.032 | -0.212 | 1.000 | -0.465 | 0.507 | 0.166 | -0.416 |
| Distance to industrial area | 0.173 | 0.019 | -0.147 | 0.115 | -0.143 | -0.030 | -0.465 | 1.000 | -0.431 | -0.011 | 0.412 |
| Distance to the nearest town | 0.040 | -0.072 | 0.186 | 0.068 | 0.049 | -0.007 | 0.507 | -0.431 | 1.000 | 0.154 | -0.465 |
| Light intensity | 0.041 | 0.018 | -0.003 | -0.096 | 0.036 | 0.028 | 0.166 | -0.011 | 0.154 | 1.000 | -0.147 |
| Moon appearance | -0.117 | 0.142 | -0.182 | 0.101 | -0.153 | -0.175 | -0.416 | 0.412 | -0.465 | -0.147 | 1.000 |

^*^ Spearman’s rho showed no important correlations between variables (correlation coefficient |rho| ≤ 0.5; Freckleton 2002).

**Reference**

Freckleton R.P. (2002) On the misuse of residuals in ecology: regression of residuals vs. multiple regression. Journal of Animal Ecology.71: 542-545.

Table B: Influence of light intensity on bat activity: AIC of each model type for each detected species.

*Selection between 4 models: GLM with Poisson distribution (Poisson), GLM with negative binomial distribution (Negative binomial), Zero inflated model with Poisson distribution (ZAP) and Zero inflated model with negative binomial distribution (ZANB).Given in bold letters are the retained models according to the smallest AIC value [45] ( / the model did not converge)*

| **Species** | **Selected model** | **Poisson** | **Negative binomial** | **ZAP** | **ZANB** |
| --- | --- | --- | --- | --- | --- |
| ***Barbastella barbastellus*** | Zero inflated model with negative binomial-count model | 1380 | 1382 | 342 | **93** |
| ***Eptesicus serotinus*** | Zero inflated model with negative binomial-count model | 1358 | 1382 | 319 | **244** |
| ***Pipistrellus pipistrellus*** | Negative binomial distribution | 2621 | **1794** | 98270 | 1798 |
| ***Pipistrellus pygmaeus*** | Zero inflated model with Poisson distribution-count model | 1119 | 873 | **67** | 69 |
| ***Pipistrellus kuhlii*** | Zero inflated model with negative binomial-count model | 2359 | 1109 | 28448 | **1103** |
| ***Pipistrellus nathusii*** | Zero inflated model with negative binomial-count model | 2513 | / | 48923 | **1385** |
| ***Nyctalus leisleri*** | Zero inflated model with negative binomial-count model | 1580 | / | 209 | **191** |
| ***Nyctalus noctula*** | Zero inflated model with Poisson distribution-count model | 1360 | / | **193** | 195 |
| ***Myotis ssp.*** | Negative binomial distribution | 2019 | **192** | 5275 | 197 |
| ***Plecotus ssp.*** | Zero inflated model with negative binomial-count model | 525 | 1271 | **97** | 99 |

**Reference**

Akaike H.. 1974. A new look at the statistical model identification". IEEE Transactions on Automatic Control 19 (6): 716–723

Table C: Influence of light type on bat activity: AIC of each model type for each detected species.

*Selection between 4 models: GLM with Poisson distribution (Poisson), GLM with negative binomial distribution (Negative binomial), Zero inflated model with Poisson distribution (ZAP) and Zero inflated model with negative binomial distribution (ZANB). Given in bold letters are the retained models according to the smallest AIC value [45] ( / the model did not converge. in bold and underlined when the AIC value is smaller than with light intensity)*

| **Species** | **Selected model** | **Poisson** | **Negative binomial** | **ZAP** | **ZANB** |
| --- | --- | --- | --- | --- | --- |
| ***Barbastella barbastellus*** | Zero inflated model with negative binomial-count model | 1243 | 2275 | / | **/** |
| ***Eptesicus serotinus*** | Zero inflated model with negative binomial-count model | 1622 | / | 281 | **259** |
| ***Pipistrellus pipistrellus*** | Negative binomial distribution | 2298 | **1541** | 71647 | 1548 |
| ***Pipistrellus pygmaeus*** | Zero inflated model with Poisson distribution-count model | 1004 | **73** | **/** | / |
| ***Pipistrellus kuhlii*** | Zero inflated model with negative binomial-count model | 2198 | 1034 | 37727 | **1033** |
| ***Pipistrellus nathusii*** | Zero inflated model with negative binomial-count model | 2252 | / | 53744 | **1258** |
| ***Nyctalus leisleri*** | Zero inflated model with negative binomial-count model | 1416 | / | 517 | **270** |
| ***Nyctalus noctula*** | Zero inflated model with Poisson distribution-count model | 1077 | / | **201** | 203 |
| ***Myotis ssp.*** | Negative binomial distribution | 1800 | **/** | / | / |
| ***Plecotus ssp.*** | Zero inflated model with negative binomial-count model | 1021 | / | **/** | / |

**Reference**

Akaike H (1974) A new look at the statistical model identification". IEEE Transactions on Automatic Control 19 (6): 716–723

Table D: Effects of light intensity, weather, spatial and landscape conditions and date on the activity of each bat species

| **Variable**  **Species – model** | **Light intensity** | **Date** | **Time after sunset** | **Temperature** | **Wind** | **Distance to**  **hedgerow** | **Semi-natural**  **area** | **Normed**  **latitude** | **Normed**  **longitude** | **Normed**  **latitude²** | **Normed**  **longitude²** |
| --- | --- | --- | --- | --- | --- | --- | --- | --- | --- | --- | --- |
| ***Barbastella barbastellus-***Zero inflated model with negative binomial-count model | -7.21(±81.06) |  | -0.02(±0.01) |  |  |  |  |  |  |  |  |
|  | F= -0.09 | NT | F= -1.48 | NT | NT | NT | NT | NT | NT | NT | NT |
|  | P= 0.93 |  | P= 0.14 |  |  |  |  |  |  |  |  |
| ***Eptesicus serotinus -*** | 6.67(±1.17) | 0.02(±0.01) | -0.01(±0.004) | -0.12(±0.09) | 0.29(±0.08) | -0.07(±0.01) | -0.006(±0.015) | -0.56(±1.0) | -2.90(±1.42) | 0.14(±0.43) | -1.82(±0.65) |
| Zero inflated model with negative binomial-count model | F=5.69 | F=1.88 | F=-2.72 | F=-1.35 | F=3.39 | F=-6.11 | F=-0.39 | F=-0.56 | F=-2.04 | F=0.33 | F=2.79 |
|  | P < 0.001 | P= 0.06 | P= 6.40.10^-3^ | P= 0.18 | P < 0.001 | P < 0.001 | P= 0.69 | P= 0.57 | P= 0.04 | P= 0.74 | P= 0.005 |
| ***Pipistrellus pipistrellus -*** | 0.07(±0.04) |  |  | -0.04(±0.06) | -0.02(±0.03) | -0.007(±0.004) | -0.03(±0.005) |  |  |  |  |
| Negative binomial distribution | F=1.9 | NT | NT | F=-0.67 | F=-0.75 | F=-1.51 | F=-6.04 | NT | NT | NT | NT |
|  | P= 0.46.10^-1^ |  |  | P= 0.50 | P= 0.45 | P= 0.13 | P < 0.001 |  |  |  |  |
| ***Pipistrellus pygmaeus-*** | 1.95(±0.21) |  |  | -0.55(0.11) |  |  | -0.01(0.008) |  |  |  |  |
| Zero inflated model with Poisson distribution-count model | F=9.43 | NT | NT | F=-5.15 | NT | NT | F=-1.38 | NT | NT | NT | NT |
|  | P < 0.001 |  |  | P < 0.001 |  |  | P= 0.17 |  |  |  |  |
| ***Pipistrellus kuhlii-*** | 0.15(±0.09) | 0.001(±0.08) | -0.01(±0.004) | -0.19(±0.09) | -0.09(±0.05) | -0.03(±0.006) | -0.01(±0.007) | -0.03(±0.39) | 0.26(±0.39) |  |  |
| Zero inflated model with negative binomial-count model | F=1.77 | F=0.16 | F=-3.16 | F=-2.1 | F=-1.75 | F=-4.62 | F=-1.85 | F=-0.07 | F=0.67 | NT | NT |
|  | P=0.76.10^-1^ | P= 0.87 | P= 1.55.10^-3^ | P= 0.03 | P=0.08 | P < 0.001 | P=0.06 | P= 0.95 | P= 0.50 |  |  |
| ***Pipistrellus nathusii-*** | 0.09(±0.07) |  |  | 0.16(0.10) | -0.14(0.03) | -0.009(0.005) |  |  |  |  |  |
| Zero inflated model with negative binomial-count model | F=1.12 | NT | NT | F=1.56 | F=-4.36 | F=-1.71 | NT | NT | NT | NT | NT |
|  | P= 0.26 |  |  | P= 0.12 | P < 0.001 | P=0.09 |  |  |  |  |  |
| ***Nyctalus leisleri-*** | -6.77 (±2.61) | 0.085(±0.04) | -0.001(±0.008) | -0.56(±0.53) | -0.14(±0.17) | 0.11(±0.03) | -0.006(±0.013) | 0.23(±0.78) | -8.0(±3.90) |  |  |
| Zero inflated model with negative binomial-count model | F=-2.58 | F=2.09 | F=-0.18 | F=-1.05 | F=-0.80 | F=4.00 | F=-4.38 | F=0.30 | F=-2.06 | NT | NT |
|  | P=9.75.10^-3^ | P= 0.04 | P= 0.86 | P= 0.29 | P= 0.42 | P < 0.001 | P < 0.001 | P= 0.76 | P= 0.04 |  |  |
| ***Nyctalus noctula-*** | 0.50 (±0.07) | -0.10(±0.01) | -0.019(±0.002) | 0.53(±0.09) | -1.43(±0.16) | 0.03(0.004) | -0.024(±0.007) | -0.09(±0.36) | -2.76(±0.64) | 5.63(±1.27) | 25.39(±2.59= |
| Zero inflated model with Poisson distribution-count model | F=6.92 | F=-8.15 | F=-6.48 | F=6.03 | F=-8.94 | F=6.07 | F=-3.35 | F=-0.27 | F=-4.29 | F=4.41 | F=9.78 |
|  | P < 0.001 | P < 0.001 | P < 0.001 | P < 0.001 | P < 0.001 | P < 0.001 | P < 0.001 | P= 0.78 | P < 0.001 | P < 0.001 | P < 0.001 |
| ***Myotis ssp.-*** | -5.98 (±2.35) |  |  | 0.13(±0.22) |  |  |  |  |  |  |  |
| Negative binomial distribution | F=-2.35 | NT | NT | F=0.59 | NT | NT | NT | NT | NT | NT | NT |
|  | P= 0.01 |  |  | P= 0.55 |  |  |  |  |  |  |  |
| ***Plecotus sp.-*** | -12.63 (±2.00) | 0.016(±0.003) | -0.021(±0.005) | 0.17(±0.03) |  |  | 0.03(±0.01) |  |  |  |  |
| Zero inflated model with Poisson distribution-count model | F=-6.18 | F=4.57 | F=-3.92 | F=4.86 | NT | NT | F=2.70 | NT | NT | NT | NT |
|  | P < 0.001 | P < 0.001 | P < 0.001 | P < 0.001 |  |  | P= 6.87.10^-3^ |  |  |  |  |

Table shows estimate values (±SE), the z-value F and the P-value P for each species. NT = not taken into account when the model did not converge

Table E: Effects of light type, weather, spatial and landscape conditions and date on the activity of each bat species

| **Variable**  **Species – model** | **Light color** | **Date** | **Time after sunset** | **Temperature** | **Wind** | **Distance to hedgerow** | **Semi-natural area** | **Normed latitude** | **Normed longitude** | **Normed latitude²** | **Normed longitude²** |
| --- | --- | --- | --- | --- | --- | --- | --- | --- | --- | --- | --- |
| ***Barbastella barbastellus-*** | White: β=-17.52 (±3.58.10^3^) | NT | NT | NT | NT | NT | NT | NT | NT | NT | NT |
| Poisson distribution | F= -0.05.10^-1^ |  |  |  |  |  |  |  |  |  |  |
|  | P =1.00 |  |  |  |  |  |  |  |  |  |  |
|  | Orange: β=-0.48 (±1.58) |  |  |  |  |  |  |  |  |  |  |
|  | F= -0.30 |  |  |  |  |  |  |  |  |  |  |
|  | P= 0.76 |  |  |  |  |  |  |  |  |  |  |
| ***Eptesicus serotinus -*** | White: β=-0.71 (±1.10) | 0.44. 10^-2^ (±0.42.10^-2^) | -0.24.10^-1^ (±00.32.10^-2^) | -0.48 (±0.08) | -0.24(±0.05) | -1.72. 10^-2^ (±0.578. 10^-2^) | -0.10 (±1.67. 10^-2^) | 9.48 (±1.34) | 5.34 (±0.80 ) | -8.90 (±1.28 ) | 6.55 (±0.95) |
| Zero inflated model with negative binomial-count model | F=-0.65 | F=1.058 | F=-7.39 | F=-6.01 | F= -5.08 | F=-2.97 | F=-5.98 | F=7.06 | F=6.69 | F=-6.96 | F=6.91 |
|  | P= 0.52 | P= 0.29 | P < 0.001 | P < 0.001 | P < 0.001 | P= 2.94.10^-3^ | P < 0.001 | P < 0.001 | P < 0.001 | P < 0.001 | P < 0.001 |
|  | Orange: β=-7.29 (±1.28) |  |  |  |  |  |  |  |  |  |  |
|  | F=-5.70 |  |  |  |  |  |  |  |  |  |  |
|  | P < 0.001 |  |  |  |  |  |  |  |  |  |  |
| ***Pipistrellus pipistrellus -*** | White: β=2.45 (±0.74) | NT | NT | 4.69. 10^-2^ (±0.07) | 0.78. 10^-2^ (±0.03) | -0.84.10^-2^ (±0.56.10^-2^) | -0.29.10^-2^ (±0.86. 10^-2^) | NT | NT | NT | NT |
| Negative binomial distribution | F= 3.32 |  |  | F= 0.67 | F= 0.22 | F= -1.48 | F= -0.34 |  |  |  |  |
|  | P < 0.001 |  |  | P= 0.48 | P= 0.81 | P= 0.11 | P= 0.72 |  |  |  |  |
|  | Orange: β= 1.72(±0.67) |  |  |  |  |  |  |  |  |  |  |
|  | F= 2.57 |  |  |  |  |  |  |  |  |  |  |
|  | P= 6.27.10^-3^ |  |  |  |  |  |  |  |  |  |  |
| ***Pipistrellus pygmaeus-*** | White: β=-14.19 (±22.28.10^2^) | NT | NT | NT | NT | NT | NT | NT | NT | NT | NT |
| Poisson distribution | F= -0.06.10^-1^ |  |  |  |  |  |  |  |  |  |  |
|  | P =0.99 |  |  |  |  |  |  |  |  |  |  |
|  | Orange: β=4.78 (±1.88) |  |  |  |  |  |  |  |  |  |  |
|  | F= 2.54 |  |  |  |  |  |  |  |  |  |  |
|  | P= 0.01 |  |  |  |  |  |  |  |  |  |  |
| ***Pipistrellus kuhlii-*** | White: β=5.33 (±1.33) | 0.80.10^-2^ (±0.94.10^-2^) | -1.02.10^-2^ (±0.33.10^-2^) | 0.12 (±0.13) | -5.41.10^-2^ (±0.07) | -0.03 (±0.75.10^-2^) | 0.05 (±0.01) | 1.32 (±0.46) | 1.26 (±0.48) | NT | NT |
| Zero inflated model with negative binomial-count model | F= 4.00 | F=0.85 | F= -3.07 | F=0.96 | F= -0.81 | F=-4.24 | F=3.23 | F= 2.87 | F=2.63 |  |  |
|  | P < 0.001 | P= 0.40 | P= 2.13.10^-3^ | P= 0.34 | P= 0.42 | P < 0.001 | P= 1.26.10^-3^ | P= 4.11.10^-3^ | P= 8.54.10^-3^ |  |  |
|  | Orange: β= 2.87 (±0.98) |  |  |  |  |  |  |  |  |  |  |
|  | F=2.91 |  |  |  |  |  |  |  |  |  |  |
|  | P= 3.56.10^-3^ |  |  |  |  |  |  |  |  |  |  |
| ***Pipistrellus nathusii-*** | White: β=0.99 (±0.73) | NT | NT | 0.14 (±0.13) | -0.13 (±0.04) | -1.5.10^-2^ (±0.72.10^-2^) | NT | NT | NT | NT | NT |
| Zero inflated model with negative binomial-count model | F= 1.37 |  |  | F=1.12 | F=-3.65 | F=-2.07 |  |  |  |  |  |
|  | P= 0.17 |  |  | P= 0.26 | P < 0.001 | P= 0.04 |  |  |  |  |  |
|  | Orange: β=0.42 (±0.56) |  |  |  |  |  |  |  |  |  |  |
|  | F= 0.75 |  |  |  |  |  |  |  |  |  |  |
|  | P= 0.46 |  |  |  |  |  |  |  |  |  |  |
| ***Nyctalus leisleri-*** | White: β=1.22 (±2.07) | -0.42.10^-2^ (±1.182.10-^2^) | -0.29.10^-2^ (±0.37.10^-2^) | 0.44(±0.20 ) | -0.16 (±0.12) | 0.43.10^-2^ (±0.01) | 0.54.10^-2^ (±0.02) | 0.43 (±0.51) | 0.86.10^-2^ (±1.16) | NT | NT |
| Zero inflated model with negative binomial-count model | F= 0.59 | F=-0.36 | F=-0.77 | F=2.23 | F= -1.29 | F=0.396 | F=0.21 | F=0.83 | F= 0.007 |  |  |
|  | P= 0.56 | P=0.72 | P= 0.44 | P= 0.02 | P= 0.20 | P= 0.69 | P= 0.83 | P= 0.40 | P= 0.99 |  |  |
|  | Orange: β=-0.97(1.47) |  |  |  |  |  |  |  |  |  |  |
|  | F= -0.66 |  |  |  |  |  |  |  |  |  |  |
|  | P < 0.001 |  |  |  |  |  |  |  |  |  |  |
| ***Nyctalus noctula-*** | White: β=-3.58 (±1.81) | -0.13 (±0.03) | -0.94.10^-2^ (±0.51.10^-2^) | 0.60 (±0.19) | -1.38 (±0.28) | 0.03 (±0.54.10^-2^) | -0.10 (±0.03) | -2.23 (±0.89) | 15.24 (±3.76) | -4.91 (±1.05) | 11.50 (±2.05) |
| Zero inflated model with Poisson distribution-count model | F= -1.98 | F= -4.50 | F= -1.84 | F=3.20 | F=-4.87 | F=5.84 | F=-3.57 | F=-2.49 | F=4.05 | F=-4.68 | F= 5.62 |
|  | P= 0.047 | P < 0.001 | P=0.06 | P= 0.001 | P < 0.001 | P < 0.001 | P < 0.001 | P= 0.01 | P < 0.001 | P < 0.001 | P < 0.001 |
|  | Orange: β=2.23 (±1.95) |  |  |  |  |  |  |  |  |  |  |
|  | F=1.14 |  |  |  |  |  |  |  |  |  |  |
|  | P= 0.25 |  |  |  |  |  |  |  |  |  |  |
| ***Myotis ssp.-*** | White: β= 3.69 (±1.80) | NT | NT | NT | NT | NT | NT | NT | NT | NT | NT |
| Poisson distribution | F= 2.05 |  |  |  |  |  |  |  |  |  |  |
|  | P =0.05 |  |  |  |  |  |  |  |  |  |  |
|  | Orange: β=0.23 (±3.66) |  |  |  |  |  |  |  |  |  |  |
|  | F= 0.06 |  |  |  |  |  |  |  |  |  |  |
|  | P= 0.95 |  |  |  |  |  |  |  |  |  |  |
| ***Plecotus sp.-*** | White: β=-17.15 (±26.79.10^2^) | NT | NT | NT | NT | NT | NT | NT | NT | NT | NT |
| Poisson distribution | F= -0.06.10^-1^ |  |  |  |  |  |  |  |  |  |  |
|  | P =0.99 |  |  |  |  |  |  |  |  |  |  |
|  | Orange: β=-1.18 (±1.95) |  |  |  |  |  |  |  |  |  |  |
|  | F= -0.61 |  |  |  |  |  |  |  |  |  |  |
|  | P= 0.54 |  |  |  |  |  |  |  |  |  |  |

Table shows estimate values (±SE). The z-value F and the P-value P for each species. NT = not taken into account when the model did not converge. β is compared to the intercept which is the absence of light.

Table F: Effects of light intensity, weather, spatial and landscape conditions and date on the activity of each group (GLM results)

| **Variable**  **Group** | **Light intensity** | **Date** | **Time after sunset** | **Temperature** | **Wind** | **Distance to**  **hedgerow** | **Semi-natural**  **area** | **Normed**  **latitude** | **Normed**  **longitude** | **Normed**  **latitude²** | **Normed**  **longitude²** |
| --- | --- | --- | --- | --- | --- | --- | --- | --- | --- | --- | --- |
| **Light-tolerant group** | 1.18 (±8.99.10^-4^) | -4.38 (±1.56.10^-4^) | -5.14 (±8.01.10^-5^) | 4.36 (±1.76.10^-4^) | -4.16.10^-2^  (±9.97.10^-4^) | -1.12.10^-2^  (±1.21.10^-4^) | -1.95.10^-2^  (±1.76.10^-4^) | -0.16 (±8.85.10^-3^) | -0.19 (±1.23.10^-2^) | 0.21 (±6.47.10^-3^) | -0.39 (±8.87.10^-3^) |
|  | F= 13.17 | F= -27.97 | F= -6.43 | F= 24.70 | F= -41.72 | F= -93.24 | F= -111.19 | F= -18.05 | F= -16.10 | F= 31.86 | F= -44.88 |
|  | P < 0.001 | P < 0.001 | P < 0.001 | P < 0.001 | P < 0.001 | P < 0.001 | P < 0.001 | P < 0.001 | P < 0.001 | P < 0.001 | P < 0.001 |
| **Light-intolerant group** | -0.47 (±0.06) | 5.93.10^-3^ (±1.69.10^-3^) | 8.59.10^-3^ (±9.26.10^-4^) | 0.39 (±0.022) | -0.25 (±0.015) | -0.015 (±1.35.10^-3^) | -2.11.10^-2^ (±1.96.10^-3^) | -2.73 (±0.19) | -1.19 (±0.18) | -0.86 (±0.11) | 0.19 (±0.16) |
|  | F= -7.70 | F= 3.50 | F= 9.28 | F= 17.82 | F= -16.96 | F= -11.22 | F= -10.75 | F= -14.14 | F= -6.58 | F= -8.09 | F= 1.24 |
|  | P < 0.001 | P < 0.001 | P < 0.001 | P < 0.001 | P < 0.001 | P < 0.001 | P < 0.001 | P < 0.001 | P < 0.001 | P < 0.001 | P=0.21 |

Table shows estimate values (±SE), the z-value F and the P-value P for each species. NT = not taken into account when the model did not converge.
